# Effects of GLP-1 receptor agonists on cardiometabolic outcomes in heart transplant recipients: A systematic review and meta-analysis of observational studies

**DOI:** 10.1016/j.jhlto.2026.100565

**Published:** 2026-04-16

**Authors:** Niti Dalal, Abhinav Saxena, Ala Mohsen, Jamil Borgi, Danielle Tatum, Aabha Divya

**Affiliations:** a Department of Epidemiology, Tulane School of Public Health and Tropical Medicine, New Orleans, LA; b East Jefferson General Hospital, Metairie, LA; c Division of Cardiothoracic Surgery, Department of Surgery, Tulane University School of Medicine, New Orleans, LA

**Keywords:** Heart transplantation, Glucagon-like peptide-1 receptor agonists, Post-transplant diabetes mellitus, Obesity, Metabolic syndrome, Meta-analysis

## Abstract

**Background:**

Cardiometabolic complications drive late morbidity and mortality after heart transplantation. Although glucagon-like peptide-1 receptor agonists (GLP-1 RAs) are established therapies for diabetes and obesity, heart transplant recipients have been excluded from pivotal trials due to concerns regarding gastrointestinal intolerance, altered drug absorption, and possible interactions with immunosuppressants. We therefore performed a systematic review and meta-analysis of observational studies to evaluate metabolic, renal, and safety outcomes associated with GLP-1 RA therapy in adult heart transplant recipients.

**Methods:**

Using a prespecified protocol registered with PROSPERO (CRD420251243172), we conducted a systematic review and meta-analysis of observational studies published in PubMed, Embase, Cochrane CENTRAL, and Scopus through January 31, 2025, evaluating GLP-1 RA therapy in adult heart transplant recipients. Two reviewers independently screened studies, extracted data, and assessed risk of bias using the ROBINS-I tool. For mixed solid-organ transplant studies, heart-transplant subgroup data were used when explicitly reported by the study authors. When heart-specific quantitative outcome data were not separately available, mixed solid-organ cohort data were described as indirect evidence and interpreted cautiously. Discrepancies were resolved through discussion, with adjudication by a third reviewer when necessary. Random-effects meta-analyses with restricted maximum likelihood estimation were performed for outcomes reported by at least 2 sufficiently similar studies.

**Results:**

Four observational studies (N=245) met inclusion criteria. Meta-analysis demonstrated significant reductions in body mass index (pooled mean difference −2.24 kg/m²; 95% CI −3.49 to −0.98; p=0.0005; I²=56.8%) and hemoglobin A1c (−0.62% points; 95% CI −0.94 to −0.31; p=0.0001; I²=0%). Secondary outcomes showed reductions in body weight (−7.82 kg; p=0.032) and LDL cholesterol (−11.88 mg/dL; p=0.034). Renal function remained stable. Reported safety data were limited, as available observational studies did not identify a consistent short-term signal of harm associated with immunosuppression management or graft outcomes.

**Conclusions:**

GLP-1 RA therapy was associated with improved weight- and glycemia-related outcomes in adult heart transplant recipients. Available observational studies did not identify a consistent signal of harm associated with immunosuppression management or graft outcomes, but the safety data were sparse and insufficient to rule out clinically important effects. Prospective trials are needed to confirm long-term cardiovascular and graft outcomes.

## Graphical abstract

**Figure:**
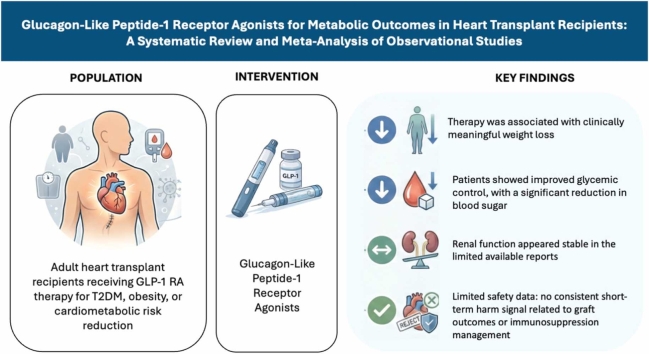

## Introduction

Cardiometabolic disease has emerged as the leading cause of death beyond the first year after heart transplantation, accounting for 30%−40% of late mortality.^1^, ^2^, ^3^ Post-transplant diabetes mellitus (PTDM) develops in 30%−40% of recipients within 12 months and confers a 2-fold increased risk of cardiac allograft vasculopathy and mortality.^1^, ^4^, ^5^ Chronic kidney disease (CKD), present in over 50% of recipients by year 5, progresses to end-stage renal disease requiring dialysis or re-transplantation in 10–20% of patients.^5^ Obesity affects an increasing proportion of recipients and contributes to hypertension, dyslipidemia, and graft dysfunction.^6^ Contemporary guidelines emphasize aggressive management of modifiable cardiometabolic risk factors as a cornerstone of long-term post-transplant care.^7^ Current therapies for PTDM often require complex regimens and may be associated with weight gain or limited weight loss.^3^ In the general population, glucagon-like peptide-1 receptor agonists (GLP-1 RAs) improve glycemic control and weight and have demonstrated cardiovascular and kidney benefits in large, randomized trials, leading to guideline endorsement for patients with type 2 diabetes, obesity, CKD, and cardiovascular disease.^8^, ^9^, ^10^, ^11^, ^12^, ^13^, ^14^, ^15^

Despite this evidence, heart transplant recipients were excluded from pivotal GLP-1 RA trials due to concerns about gastrointestinal intolerance, altered absorption, and potential interactions with calcineurin inhibitors.^16^, ^17^, ^18^, ^19^ The extent to which GLP-1 RAs may affect tacrolimus or cyclosporine absorption, immunosuppression adequacy, or rejection risk remains incompletely characterized in the transplant population. Small studies in kidney and liver transplantation suggest GLP-1 RAs may improve metabolic parameters without destabilizing calcineurin-inhibitor levels or increasing rejection risk, but these findings cannot be directly extrapolated to heart transplant recipients.^19^, ^20^, ^21^, ^22^ However, heart transplant recipients differ fundamentally due to their unique hemodynamics, higher rejection vigilance, and different immunosuppression intensity, precluding direct extrapolation. No comprehensive systematic review and meta-analysis have specifically evaluated the effects of GLP-1 RAs in adult heart transplant recipients. We hypothesized that GLP-1 RA therapy would be associated with improved cardiometabolic profiles. The objective of this systematic review and meta-analysis was to consolidate available evidence regarding the metabolic, renal, and safety outcomes of GLP-1 RA therapy in adult heart transplant recipients.

## Methods

### Study design and reporting standards

This systematic review and meta-analysis followed the Preferred Reporting Items for Systematic Reviews and Meta-Analyses (PRISMA) 2020 guidelines.^23^ A prespecified protocol was registered with PROSPERO (CRD420251243172) before study initiation.

### Eligibility criteria

#### Population

Adults (≥18 years) who underwent orthotopic heart transplantation and received any GLP-1 receptor agonist post-transplant for type 2 diabetes, obesity (BMI ≥30 kg/m²), or cardiometabolic risk reduction.

#### Outcomes

Primary outcomes were change in body mass index (BMI, kg/m²) and hemoglobin A1c (HbA1c, %) from baseline to ≥3 months follow-up. Secondary outcomes included body weight, low-density lipoprotein cholesterol (LDL-C), triglycerides, estimated glomerular filtration rate (eGFR), and insulin requirements. Safety outcomes included acute rejection, calcineurin inhibitor dose adjustments, gastrointestinal adverse events, and treatment discontinuations.

#### Study design

We included observational comparative cohorts, case-control studies, and single-group pre-post intervention studies with ≥5 participants reporting quantitative outcomes. Randomized trials were eligible a priori, but none met the inclusion criteria. Exclusions included case reports, studies without extractable heart transplant data, and non-English language publications. Mixed solid-organ transplant studies including heart transplant recipients were eligible but only used as indirect evidence unless they reported heart-specific data.

### Search strategy

A comprehensive literature search was conducted in PubMed/MEDLINE, Embase, Cochrane CENTRAL, and Scopus from inception through January 31, 2025. Search strategies incorporated controlled vocabulary (MeSH, Emtree) and keywords including: (GLP-1 OR semaglutide OR liraglutide OR dulaglutide OR tirzepatide OR exenatide) AND (heart transplant OR cardiac transplant). The reference lists of included studies and relevant systematic reviews were screened using backward and forward citation tracking.

### Study selection and data extraction

**Figure 1:**
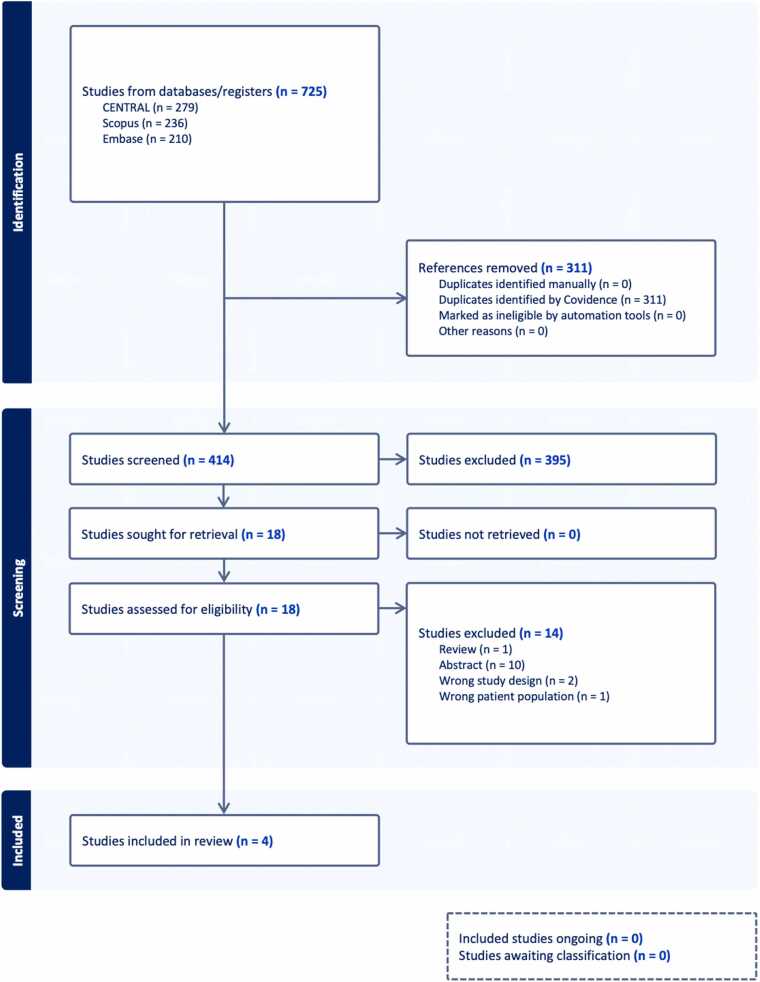
PRISMA 2020 flow diagram of study selection for the systematic review.

Two reviewers independently screened titles, abstracts, and full texts, with discrepancies resolved through discussion or third-reviewer adjudication. The selection process is summarized in a PRISMA flow diagram (Figure 1). Data were extracted using a standardized form that included study design, sample size, participant characteristics, GLP-1 receptor agonist type and dose, follow-up duration, co-interventions/confounders (including concomitant SGLT2 inhibitor exposure when reported), and outcomes. For mixed solid-organ transplant studies, only outcomes explicitly reported for heart transplant recipients were extracted for heart-specific synthesis. If an endpoint could not be isolated to the heart-transplant subgroup, that study contributed only to the qualitative review for that endpoint. For continuous outcomes, mean changes and standard deviations (or 95% CIs) were extracted. When unavailable, standard deviations were calculated from CIs, standard errors, or t-statistics.

### Risk of bias assessment

Two reviewers independently assessed risk of bias using the ROBINS-I tool because all included studies were non-randomized (Table 3). Domains included confounding, selection, intervention classification, missing data, outcome measurement, and selective reporting. Disagreements were resolved through discussion or third-reviewer adjudication.

**Table 1:** Characteristics of Included Studies

Study	Year	Country	Design	N	Population	GLP-1 RA Agent (s) and Dose	Follow-up Duration	Outcomes Reported
Donald et al.	2024	USA	Pre-post single-group	74	HT recipients; 81% T2DM; mean age 55.5 years; 28% female	Semaglutide (76%; mean 0.88 mg), liraglutide, dulaglutide, tirzepatide	Median 383 days (∼12 months)	BMI, HbA1c, weight, LDL, TG, eGFR, insulin dose
Sammour et al.	2021	USA	Pre-post single-group	21	HT recipients with obesity/T2DM; mean age 59.4 years; 67% male	Liraglutide (primarily)	Median 9.1 months (∼6-12 months)	BMI, weight, HbA1c, LDL
Thiyagarajan et al.	2020	USA	Pre-post single-group	19	Mixed solid-organ recipients: HT,^5^ kidney,^7^ liver^7^; median age 62 years	Liraglutide,^10^ dulaglutide,^5^ others^4^	12 months	BMI, HbA1c, weight, LDL, eGFR
Dotan et al.	2024	Israel	Matched retrospective cohort (169 GLP-1 RA users:169 controls)	338	Mixed solid-organ recipients; 80% kidney; mean age 58 years; 69% male	Liraglutide (34%), dulaglutide (46%), semaglutide (20%)	Median 3.1 years	BMI, HbA1c, LDL, TG (not pooled)

Abbreviations: BMI, body mass index; eGFR, estimated glomerular filtration rate; GLP-1 RA, glucagon-like peptide-1 receptor agonist; HbA1c, glycated hemoglobin; HT, heart transplant; LDL, low-density lipoprotein cholesterol; TG, triglycerides; T2DM, type 2 diabetes mellitus.

For mixed solid-organ transplant studies, only heart-transplant subgroup data explicitly reported by the original authors were used for heart-specific synthesis; outcomes that could not be separated for heart recipients were not pooled. Follow-up duration is reported as provided in the original studies; approximate conversions are shown for readability where applicable. The matched cohort study by Dotan et al. was included in the qualitative synthesis but was not pooled with single-group pre-post studies because of methodological heterogeneity.

**Table 2:** Pooled Results from Random-Effects Meta-analyses

Outcome	Studies (k)	Participants (N)	Pooled MD (95% CI)	I² (%)	p-heterogeneity	p-overall
BMI (kg/m²)	3	114	-2.24 (−3.49 to −0.98)	56.8	0.106	<0.001
HbA1c (%)	2	93	-0.62 (−0.94 to −0.31)	0.0	0.736	<0.001
Weight (kg)	2	40	-7.82 (−14.99 to −0.65)	71.3	0.062	0.032
LDL-C (mg/dL)	2	93	-11.88 (−22.85 to −0.91)	36.6	0.209	0.034

Abbreviations: CI, confidence interval; HbA1c, glycated hemoglobin; k, number of studies; LDL-C, low-density lipoprotein cholesterol; MD, mean difference.

Footnotes: Pooled estimates were derived from single-group pre-post change-from-baseline analyses using random-effects REML models. The matched cohort study by Dotan et al. was not pooled with pre-post studies because of methodological heterogeneity.

Individual study data used for pooling: BMI - Donald et al. −1.80 +/- 6.70 (n=74); Sammour et al. −4.00 +/- 4.72 (n=21); Thiyagarajan et al. −1.63 +/- 2.00 (n=19). HbA1c - Donald et al. −0.60 +/- 1.50 (n=74); Thiyagarajan et al. −0.75 +/- 1.78 (n=19). Weight - Sammour et al. −12.34 +/- 17.04 kg (n=21); Thiyagarajan et al. −4.86 +/- 6.49 kg (n=19). LDL-C - Donald et al. −8.3 +/- 34.7 mg/dL (n=74); Thiyagarajan et al. −20.63 +/- 39.0 mg/dL (n=19).

Leave-one-out sensitivity analyses showed preserved direction and statistical significance of the pooled estimates.

**Table 3:** ROBINS-I Risk of Bias Summary

Domain / Study	Confounding	Selection	Intervention Classification	Deviations	Missing Data	Outcome Measurement	Selective Reporting	Overall
Donald 2024	Serious	Moderate	Low	Serious	Low	Low	Low	Serious
Sammour 2021	Serious	Serious	Moderate	Serious	Low	Moderate	Low	Serious
Thiyagarajan 2020	Serious	Moderate	Low	Serious	Low	Low	Low	Serious
Dotan 2024 (matched)	Moderate	Low	Low	Moderate	Low	Low	Low	Moderate

Abbreviations: ROBINS-I, Risk Of Bias In Non-randomized Studies of Interventions.

Footnote: Confounding judgments were driven by non-random treatment allocation, lack of concurrent controls in pre-post studies, baseline metabolic severity, time from transplant, steroid exposure, changes in immunosuppression, and concomitant glucose-lowering therapies including SGLT2 inhibitors when reported.

### Statistical analysis

Meta-analyses were performed using the metafor package (version 4.8–0) in R (version 4.5.0). Random-effects models with restricted maximum likelihood (REML) estimation were used for outcomes reported by ≥2 studies with sufficiently similar heart-transplant-specific outcome definitions. Continuous outcomes were summarized as mean differences with 95% CIs. Pre-post analyses used change-from-baseline estimates. Between-group comparisons from comparative cohorts were not pooled with pre-post data. Statistical heterogeneity was quantified using the I² statistic and the Cochran Q test, p < 0.10 indicated significant heterogeneity. Leave-one-out sensitivity analyses were conducted for all pooled outcomes to assess the influence of individual studies on effect estimates. Prespecified subgroup analyses were planned but not performed because of the limited number of studies. Sparse safety data, including rejection events and immunosuppressant dose changes, were synthesized descriptively rather than quantitatively. Because one included study reported pooled outcomes for a mixed solid-organ cohort that included heart transplant recipients, these estimates were considered indirect evidence when incorporated into the synthesis. Formal assessment of publication bias using funnel plots and Egger's regression test was not performed because fewer than 10 studies were available for each pooled outcome, limiting statistical power for bias detection. Sex was reported descriptively; sex-stratified analyses were not feasible due to limited data.

### Certainty of evidence

The Grading of Recommendations Assessment, Development and Evaluation (GRADE) framework was used to assess the certainty of evidence for each primary and key secondary outcome. Each outcome was rated as high, moderate, or low certainty based on five domains: risk of bias, imprecision, inconsistency, indirectness, and publication bias (Table 4).

**Table 4:** GRADE Evidence Certainty Assessment

Outcome	Studies (N)	Effect Estimate (95% CI)	Risk of Bias	Inconsistency	Indirectness	Imprecision	Overall Certainty	Rationale for Downgrading
BMI reduction	3 (114)	-2.24 kg/m² (−3.49 to −0.98)	Serious^a^	Not serious^b^	Not serious	Not serious	⊕⊕⊕◯ MODERATE	Observational design without randomization; consistent direction across studies.
HbA1c reduction	2 (93)	-0.62% (−0.94 to −0.31)	Serious^a^	Not serious^c^	Not serious	Not serious	⊕⊕⊕◯ MODERATE	Observational design; zero heterogeneity (I² = 0%); precise estimates.
Weight reduction	2 (40)	-7.82 kg (−14.99 to −0.65)	Serious^a^	Serious^d^	Not serious	Serious^e^	⊕⊕◯◯ LOW	Observational design; substantial heterogeneity (I² = 71.3%); wide confidence intervals.
LDL-C reduction	2 (93)	-11.88 mg/dL (−22.85 to −0.91)	Serious^a^	Not serious	Not serious	Serious^e^	⊕⊕◯◯ LOW	Observational design; wide confidence intervals; small sample.
eGFR stability	1 (74)	No significant change	Serious^a^	Not assessable^f^	Not serious	Very serious^g^	⊕◯◯◯ VERY LOW	Single observational study; short follow-up; no meta-analysis possible.

GRADE certainty ratings reflect outcome-level assessment and were downgraded for study design, inconsistency, and/or imprecision as appropriate.

Certainty levels: ⊕⊕⊕⊕ High = very confident; ⊕⊕⊕◯ Moderate = moderately confident; ⊕⊕◯◯ Low = limited confidence; ⊕◯◯◯ Very low = very little confidence.

a Pre-post observational designs without randomization or blinding; risk of selection bias, temporal confounding, and regression to the mean.

b I² = 56.8% (moderate) but effect direction was consistent and sensitivity analysis was robust.

c I² = 0%; effect direction was consistent.

d I² = 71.3%; likely driven by cohort differences.

e Confidence intervals were wide and approached the null.

f Single study precluded heterogeneity assessment.

g Single study, short follow-up (12 months), and no between-group comparison.

## Results

### Study selection

The systematic search identified 725 records; 414 unique records remained after deduplication. Following title and abstract screening, 18 full-text articles underwent detailed assessment. Fourteen studies were excluded: 10 were conference abstracts without sufficient quantitative data, two had inappropriate study designs, one included an ineligible population, and one was a narrative review. Four studies met the inclusion criteria and were included in the qualitative synthesis (Figure 1).

### Study characteristics

The included studies comprised three single-group pre–post cohorts^24^, ^25^, ^26^ and one matched retrospective cohort study.^20^ Studies were published between 2020 and 2024 and conducted in the United States (n = 3) and Israel (n = 1). Median follow-up ranged from 9 to 37 months. Two studies included mixed solid-organ transplant populations. For these studies, only heart-transplant subgroup data explicitly reported by the original authors were extracted for heart-specific summaries. Outcomes that could not be separated for heart recipients were excluded from pooled analyses. One study also included concomitant SGLT2 inhibitor exposure, which was captured as an important co-intervention and considered in the risk-of-bias assessment and interpretation.

Among pre–post studies, GLP-1 receptor agonists were initiated a median of 3–5 years following heart transplantation. Semaglutide was the most frequently prescribed agent, followed by liraglutide and dulaglutide. Baseline prevalence of type 2 diabetes ranged from 60% to over 80%, and mean baseline body mass index ranged from 31 to 36 kg/m² (Table 1).

### Risk of Bias

All included studies were observational and assessed using the ROBINS-I tool. Overall risk of bias ranged from moderate to serious, driven primarily by confounding, selection bias, absence of concurrent controls in pre-post studies, and co-interventions. Important potential confounders included baseline obesity and diabetes severity, time from transplant, concurrent glucose-lowering therapies such as SGLT2 inhibitors, steroid exposure, and changes in immunosuppression. The matched cohort study demonstrated lower overall risk of bias but remained susceptible to residual confounding despite propensity score matching (Table 3).

### Primary outcomes

#### Body mass index

Three pre-post studies reported BMI change data. Random-effects meta-analysis (k = 3) demonstrated a statistically significant reduction in BMI following GLP-1 RA initiation:

*Pooled mean difference: −2·24 kg/m² (95% CI −3·49 to −0·98; p = 0·0005)*

Moderate heterogeneity was observed (I² = 56.8%; Q (2) = 4.48, p = 0.106). Leave-one-out sensitivity analyses confirmed the robustness of the pooled estimate, with consistent direction and statistical significance across analyses. The observed heterogeneity appeared driven by differences in baseline obesity severity, GLP-1 receptor agonist selection, and follow-up duration.

**Figure 2:**
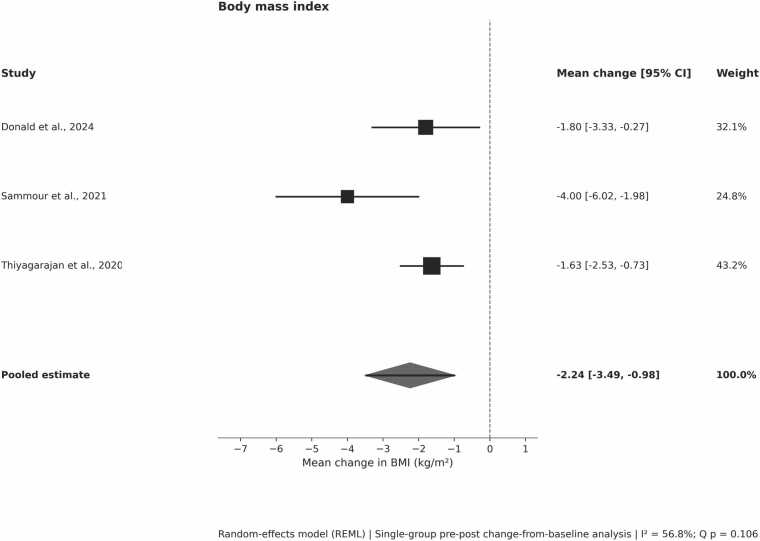
Effects of GLP-1 receptor agonists on body mass index after heart transplantation. Pooled estimates were generated using a random-effects model. Negative values indicate reductions in BMI.

The matched cohort study reported a smaller, non-significant between-group difference in BMI. This finding was not pooled due to methodological heterogeneity but was directionally consistent (Figure 2).

**Figure 3:**
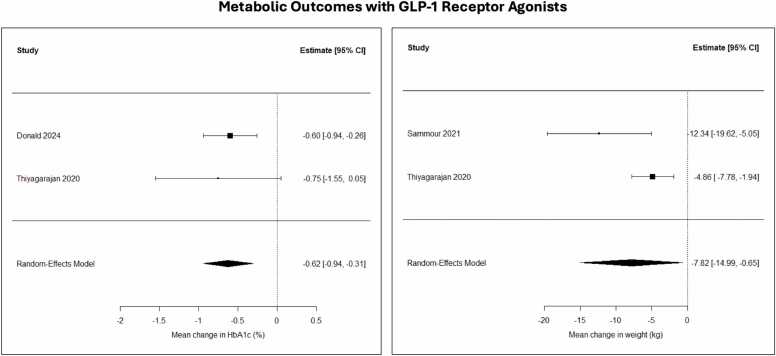
Effects of GLP-1 receptor agonists on metabolic outcomes after heart transplantation. (A) Mean change in HbA1c (%). (B) Mean change in body weight (kg). Pooled estimates were generated using random-effects models. Negative values indicate reductions from baseline.

#### Glycemic control

Two pre-post studies reported HbA1c outcome data. Meta-analysis (k = 2) demonstrated a statistically significant reduction in HbA1c:

*Pooled mean difference: −0·62% points (95% CI −0·94 to −0·31; p = 0·0001)*

No heterogeneity was observed (I² = 0%; Q (1) = 0.11, p = 0.736), and sensitivity analyses demonstrated stable effect estimates. The matched cohort study similarly reported a significant between-group reduction in HbA1c favoring GLP-1 receptor agonist users.

### Secondary outcomes

#### Body weight

Two pre-post studies reported absolute body weight change. Random-effects meta-analysis (k = 2) demonstrated a statistically significant weight reduction*:*

*Pooled mean difference: −7·82 kg (95% CI −14·99 to −0·65; p = 0·032)*

Substantial heterogeneity was observed (I² = 71.3%; Q (1) = 3.49, p = 0.062), likely reflecting differences in baseline obesity, GLP-1 receptor agonist agent and dosing, duration of therapy, and intensity of concurrent lifestyle interventions. Despite heterogeneity, the direction of effect was consistent across studies.

#### Low-density lipoprotein cholesterol

Two pre-post studies reported LDL-C change. Meta-analysis (k = 2) demonstrated a statistically significant reduction:

*Pooled mean difference: −11·88 mg/dL (95% CI −22·85 to −0·91; p = 0·034)*

Low-to-moderate heterogeneity was observed (I² = 36.6%; Q (1) = 1.58, p = 0.209). The matched cohort study did not demonstrate a significant between-group difference in LDL cholesterol.

Triglyceride outcomes were inconsistently reported and were not pooled. One study demonstrated a non-significant pre–post reduction, while the matched cohort study reported a significant between-group reduction favoring GLP-1 receptor agonist therapy.

#### Renal function by estimated glomerular filtration rate (eGFR)

Renal outcomes were reported in a limited number of studies. One pre–post study evaluating estimated glomerular filtration rate (eGFR) showed no significant change following initiation of a GLP-1 receptor agonist over approximately 12 months. Additional studies reported stable renal function, with no evidence of acute kidney injury or accelerated decline. Due to limited data and heterogeneous reporting, a meta-analysis of renal outcomes was not performed.

#### Insulin requirements

The magnitude of insulin reduction varied substantially, likely reflecting differences in baseline insulin resistance, concurrent medication use, and dietary interventions.

### Safety outcomes

#### Immunosuppression management and graft rejection

Safety data related to immunosuppression and graft outcomes were limited and inconsistently reported across studies. Tacrolimus levels were generally reported as stable when monitored after GLP-1 receptor agonist initiation. A small proportion of patients required calcineurin inhibitor dose adjustments, though none could be clearly attributable to initiation of GLP-1 receptor agonist. No study reported a clear increase in acute or chronic rejection temporally associated with GLP-1 receptor agonist therapy, although these events were sparsely reported and not systematically assessed. Also, rejection events were rare in the available reports. One acute rejection event was reported temporally following initiation in a single patient, though causality could not be established.

#### Adverse events

Gastrointestinal intolerance was the most commonly reported adverse event and accounted for the majority of treatment discontinuations. Overall discontinuation rates ranged from approximately 5% to 15%, consistent with rates observed in non-transplant populations. No study reported episodes of diabetic ketoacidosis, acute pancreatitis, or severe hypoglycemia directly attributable to GLP-1 RA therapy. No cases of acute kidney injury or sudden eGFR decline were documented. Thiyagarajan et al. evaluated thyroid nodules and malignancy surveillance but found no safety signals.

### Certainty of evidence assessment

GRADE assessments evaluated the quality of evidence across five domains. Using the GRADE framework, certainty of evidence was rated as moderate for reductions in BMI and HbA1c, downgraded primarily due to observational study design.^27^ Certainty was rated as low for body weight and LDL cholesterol outcomes due to heterogeneity and imprecision, and very low for renal outcomes due to limited data. No outcomes were downgraded for indirectness, as all were directly relevant to post-transplant care, or for publication bias, as assessment was not feasible with <10 studies^27^, ^28^ (Table 4).

## Discussion

This systematic review and meta-analysis is, to our knowledge, the first to synthesize the available evidence in adult heart transplant recipients. Across a small and methodologically limited observational literature, our findings demonstrate that GLP-1 RA therapy is associated with clinically meaningful improvements in cardiometabolic parameters. These findings align with the established efficacy of GLP-1 RAs in non-transplant populations.^8^, ^9^, ^10^, ^11^, ^12^, ^29^ However, conclusions regarding graft-related safety and immunosuppressant interactions should be drawn with caution, as these outcomes were reported inconsistently.

### Key findings

GLP-1 RA therapy was associated with significant reductions in BMI (pooled mean −2.24 kg/m²) and HbA1c (−0.62% points), corresponding to approximately 7–10 kg weight loss in the post-transplant setting. Even modest weight reduction has been associated with improvements in blood pressure, insulin sensitivity, and functional capacity in transplant recipients, and may mitigate the progression of obesity-related complications.^2^, ^3^, ^6^, ^7^, ^30^, ^31^

It also resulted in consistent and significant improvement in glycemic control, with a pooled reduction in hemoglobin A1c of 0.62% points and no observed heterogeneity. This magnitude of glycemic improvement is comparable to that observed in non-transplant populations and is relevant given the high prevalence of post-transplant diabetes mellitus and its association with adverse cardiovascular and graft-related outcomes.^1^, ^3^, ^5^ Several studies reported reduced insulin requirements, with some patients discontinuing insulin entirely.^8^, ^9^, ^10^, ^32^, ^33^ Reported safety outcomes were limited. Across the included studies, tacrolimus levels generally remained stable when monitored, and some patients required calcineurin inhibitor dose adjustments. However, these reports were based on small sample sizes, were not uniformly collected, and could not establish causality. Gastrointestinal intolerance and treatment discontinuation were the most consistently described adverse effects and were similar to those observed in non-transplant populations.^8^, ^9^, ^10^, ^11^, ^12^, ^34^ The metabolic benefits observed appear comparable to those in non-transplant populations, though absolute reductions were smaller, likely reflecting the transplant population's baseline comorbidity burden and physical activity limitations.^5^, ^6^, ^11^, ^13^

Emerging data from kidney and liver transplant recipients similarly show metabolic improvements without an obvious signal of immunosuppression destabilization, although those studies are likewise observational.^20^, ^21^ The present analysis extends these observations to heart transplant recipients, and should be viewed as hypothesis-generating rather than definitive. The consistency of glycemic and weight effects across transplant and non-transplant populations suggests that the efficacy of GLP-1 RAs is not meaningfully attenuated by immunosuppressive therapy. However, the observational nature of transplant studies precludes definitive comparison to randomized controlled trials.

### Safety and immunosuppression considerations

A key concern among transplant recipients is whether GLP-1 RAs alter the absorption of concurrent immunosuppressants due to delayed gastric emptying.^16^, ^17^ In the heart-transplant studies included here, evidence addressing this question was limited to small observational reports. 5–7% of transplant recipients in observational studies required dose adjustments attributable to initiation of GLP-1 RA. Tacrolimus levels remained generally stable across studies, and dose changes could have reflected routine clinical adjustment rather than a drug-drug interaction. Gastrointestinal adverse events led to discontinuation in approximately 5–10% of patients, consistent with discontinuation rates in non-transplant populations receiving GLP-1 RA therapy.

Likewise, because rejection events were rare and follow-up was limited, the current literature is insufficient to exclude modest but clinically meaningful effects on rejection risk or graft function. Accordingly, our findings should not be interpreted as proving immunologic safety. Rather, the available studies did not identify a consistent signal of frequent short-term harm, while remaining underpowered to detect uncommon graft-related events. Longer-term prospective studies with prespecified monitoring of tacrolimus exposure, rejection, and graft function are required before firm conclusions can be drawn.

### Renal outcomes

Renal dysfunction is highly prevalent after heart transplantation and represents a major determinant of long-term morbidity and mortality.^4^, ^5^ In the present analysis, renal outcomes were reported inconsistently and could not be pooled. Available data demonstrated a stable estimated glomerular filtration rate following GLP-1 RA initiation, with no signal for accelerated decline or acute kidney injury. While large trials in non-transplant populations have demonstrated renoprotective effects, current evidence in heart transplant recipients supports renal safety rather than benefit. Dedicated prospective studies with kidney-specific endpoints are needed.

### Clinical implications

Despite the observational nature of the available data, these findings have important implications for clinical practice. GLP-1 receptor agonists appear to be a reasonable therapeutic option for stable heart transplant recipients with post-transplant diabetes mellitus, obesity, or inadequate glycemic control on conventional therapies. Given the observed metabolic benefits and the absence of a consistent short-term harm signal in limited observational data, GLP-1 RAs may be considered in selected stable heart transplant recipients with careful clinical and immunosuppressant monitoring. Patients most likely to benefit may include those who are more than one-year post-transplant with stable graft function, established diabetes or obesity, and preserved renal function. Agent selection should be individualized based on indication, tolerability, dosing frequency, and insurance coverage.

### Knowledge gaps and future directions

Prospective randomized controlled trials are needed to confirm the metabolic benefits of GLP-1 RAs in heart transplant recipients and to evaluate effects on cardiovascular events, cardiac allograft vasculopathy, graft survival, and mortality. Longer-term follow-up studies are essential to assess the durability of benefit and late safety signals.

Prospective pharmacokinetic studies evaluating interactions between GLP-1 RAs and immunosuppressive agents would provide mechanistic insight and inform optimal dosing strategies. Future studies should also explore whether treatment effects vary by GLP-1 RA agent, time since transplant, baseline renal function, or degree of metabolic disease. Finally, real-world implementation studies examining adherence, cost-effectiveness, and patient-reported outcomes will be critical to guide widespread adoption in transplant care.

## Limitations

All included studies were observational, with inherent susceptibility to selection bias, confounding by indication, and co-intervention bias. Pre-post designs lacked control groups, and residual confounding cannot be excluded. Two studies included mixed solid-organ transplant populations, and our review relied on heart-transplant subgroup data only when these were explicitly reported; incomplete subgroup reporting may have limited certainty for some endpoints. One study included concomitant exposure to an SGLT2 inhibitor, which may have contributed to the observed metabolic changes. Sample sizes were small, follow-up was limited, and outcome reporting was heterogeneous. Publication bias assessment was underpowered. Certainty of evidence was moderate for BMI and HbA1c. Safety outcomes were sparsely reported and not suitable for pooling. Data regarding weight rebound after discontinuation of GLP-1 RA therapy were not systematically reported. Sex-specific analyses were not feasible.

## Conclusion

In this systematic review and meta-analysis, GLP-1 receptor agonist therapy was associated with improved weight and glycemic control in adult heart transplant recipients. Renal function appeared stable in the limited available reports. Current observational data did not identify a consistent short-term signal of harm related to immunosuppression management or graft outcomes, but the evidence is insufficient to establish safety with confidence. These findings suggest that GLP-1 RAs may represent a potentially useful adjunctive therapy for cardiometabolic management after heart transplantation. Prospective, adequately powered trials are needed before GLP-1 RAs can be definitively recommended as standard-of-care therapy in this population.

## Ethics and transplant declaration

This study is a systematic review and meta-analysis of previously published data. All included studies were conducted in accordance with institutional and national ethical standards and complied with the Declaration of Helsinki. The authors affirm that no organs or tissues were procured from executed prisoners or prisoners of conscience, and that donor consent and organ procurement procedures adhered to internationally accepted ethical guidelines, including the Declaration of Istanbul and ISHLT transplant ethics policies.

## Financial disclosure / Conflict of interest statement

None.

## Declaration of Generative AI and AI-assisted technologies in the writing process

AI-assisted tools were used solely for language editing and grammatical refinement. No AI tools were used for data analysis, result interpretation, or the generation of scientific content. All authors reviewed and approved the final manuscript and take full responsibility for its accuracy and integrity.

## Declaration of Competing Interest

The authors declare that they have no known competing financial interests or personal relationships that could have appeared to influence the work reported in this paper.
