# Supplementary material for: Effects of GLP-1 receptor agonists on cardiometabolic outcomes in heart transplant recipients: A systematic review and meta-analysis of observational studies
Source: JHLT Open. 2026 Apr 16;13:100565. doi: 10.1016/j.jhlto.2026.100565 (PMC13137046; doi:10.1016/j.jhlto.2026.100565)
Supplement: Supplementary file 1 — Supplemental material [file mmc1.docx]

**Supplementary Table S1. Study-level extracted data used for quantitative synthesis**

| **Outcome** | **Study** | **Design / subgroup used** | **n** | **Extracted effect measure** | **Dispersion** | **Notes for synthesis** |
| --- | --- | --- | --- | --- | --- | --- |
| BMI (kg/m²) | Donald et al., 2024 | Pre-post HT cohort | 74 | Mean change = -1.80 | SD = 6.70 | Included in pooled pre-post meta-analysis |
| BMI (kg/m²) | Sammour et al., 2021 | Pre-post HT cohort | 21 | Mean change = -4.00 | SD = 4.72 | Included in pooled pre-post meta-analysis |
| BMI (kg/m²) | Thiyagarajan et al., 2020 | Pre-post mixed solid-organ cohort; heart-specific data used as reported | 19 | Mean change = -1.63 | SD = 2.00 | Included in pooled pre-post meta-analysis |
| BMI (kg/m²) | Dotan et al., 2024 | Matched cohort, mixed solid-organ recipients | 169 vs 169 | Between-group change = -0.40 | SD = 2.35 | Not pooled owing to methodological heterogeneity and limited heart-specific subgroup reporting |
| HbA1c (%) | Donald et al., 2024 | Pre-post HT cohort | 74 | Mean change = -0.60 | SD = 1.50 | Included in pooled pre-post meta-analysis |
| HbA1c (%) | Thiyagarajan et al., 2020 | Pre-post mixed solid-organ cohort; heart-specific data used as reported | 19 | Mean change = -0.75 | SD = 1.78 | Included in pooled pre-post meta-analysis |
| HbA1c (%) | Dotan et al., 2024 | Matched cohort, mixed solid-organ recipients | 169 vs 169 | Between-group change = -0.40 | SD = 1.02 | Reported qualitatively only; not pooled |
| Body weight (kg) | Sammour et al., 2021 | Pre-post HT cohort | 21 | Mean change = -12.34 | SD = 17.04 | Included in pooled pre-post meta-analysis; converted from 27.2 lb reduction |
| Body weight (kg) | Thiyagarajan et al., 2020 | Pre-post mixed solid-organ cohort; heart-specific data used as reported | 19 | Mean change = -4.86 | SD = 6.49 | Included in pooled pre-post meta-analysis |
| LDL-C (mg/dL) | Donald et al., 2024 | Pre-post HT cohort | 74 | Mean change = -8.30 | SD = 34.70 | Included in pooled pre-post meta-analysis |
| LDL-C (mg/dL) | Thiyagarajan et al., 2020 | Pre-post mixed solid-organ cohort; heart-specific data used as reported | 19 | Mean change = -20.63 | SD = 39.00 | Included in pooled pre-post meta-analysis |
| LDL-C (mg/dL) | Dotan et al., 2024 | Matched cohort, mixed solid-organ recipients | 169 vs 169 | Between-group change = -0.90 | SD = 30.50 | Reported qualitatively only; not pooled |
| Triglycerides (mg/dL) | Donald et al., 2024 | Pre-post HT cohort | 74 | Mean change = -10.30 | SD = 70.30 | Not pooled because of heterogeneous reporting/design |
| Triglycerides (mg/dL) | Dotan et al., 2024 | Matched cohort, mixed solid-organ recipients | 169 vs 169 | Between-group change = -23.00 | SD = 79.40 | Not pooled because of heterogeneous reporting/design |
| eGFR (mL/min/1.73 m²) | Donald et al., 2024 | Pre-post HT cohort | 74 | Baseline 57.2 to follow-up 56.1 | p = 0.588 | No significant change; no meta-analysis possible |

Abbreviations: BMI, body mass index; eGFR, estimated glomerular filtration rate; HbA1c, glycated hemoglobin; HT, heart transplant; LDL-C, low-density lipoprotein cholesterol; SD, standard deviation.

Pooled meta-analyses were based on single-group pre-post change-from-baseline estimates using random-effects REML models.

For mixed solid-organ studies, only heart-transplant subgroup data explicitly reported by the original authors were used for heart-specific synthesis; outcomes that could not be isolated to heart recipients were not pooled.

Dotan et al. was included in the qualitative synthesis but not pooled with single-group pre-post studies because of methodological heterogeneity and limited heart-specific subgroup reporting.
